# Supplementary figures and images for: Taxonomic and functional components of avian metacommunity structure along an urban gradient
Source: PLoS One. 2022 Aug 9;17(8):e0271405. doi: 10.1371/journal.pone.0271405 (PMC9362948; doi:10.1371/journal.pone.0271405)

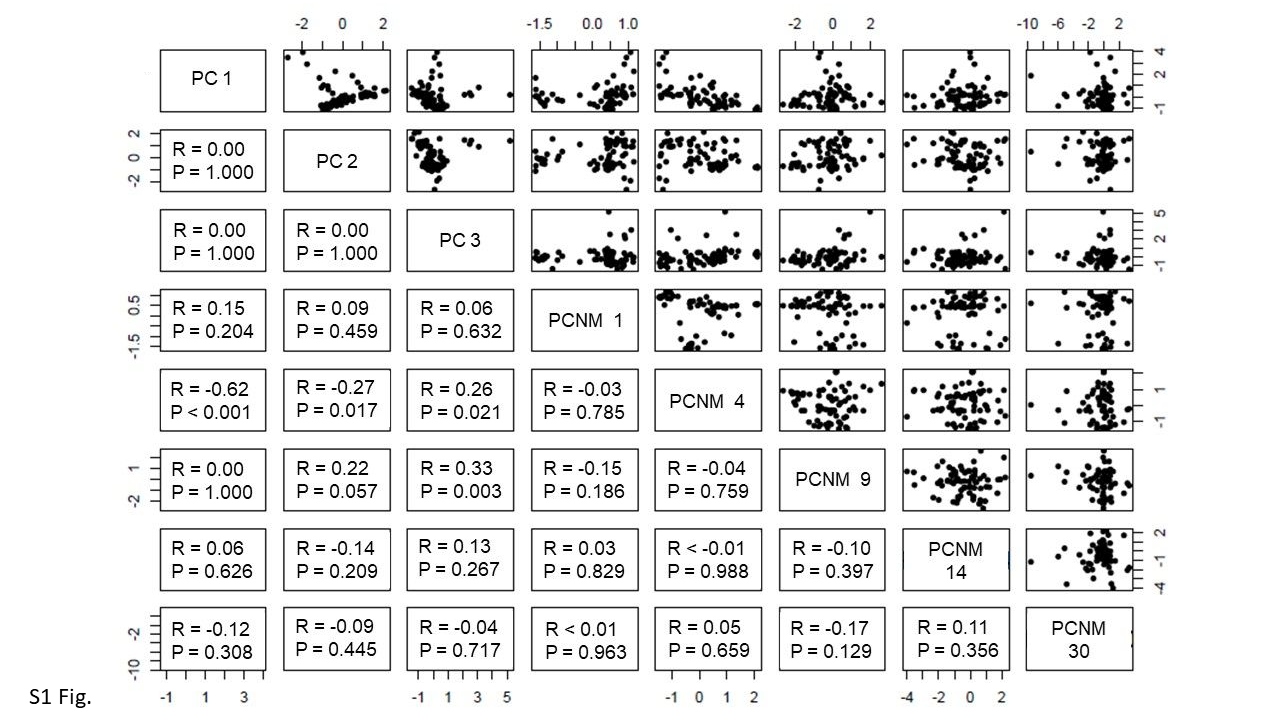

Supplement: S1 Fig — (TIF) [file pone.0271405.s001.tif]
