# Supplementary material for: Taxonomic and functional components of avian metacommunity structure along an urban gradient
Source: PLoS One. 2022 Aug 9;17(8):e0271405. doi: 10.1371/journal.pone.0271405 (PMC9362948; doi:10.1371/journal.pone.0271405)
Supplement: S1 Table — Canonical correspondence analysis was conducted on species occurrences and environmental and spatial variables in Texas from May to August from 2013 to 2017. Loadings from canonical correspondence analysis for avian species responses to environmental and spatial variables are presented. This table corresponds to Fig 3A, 3B of the 3C, the first axis of the CCA was positively associated with PC 3, and the second axis to PC2 and to a lesser extent negatively associated with PC 1. For species responses to spatial structures, the first axis of CCA was positively associated with PCNM 1 and negatively associated with PCNM 14 and 30. The second axis was positively associate with PCNM 9 and 14 and negatively associated with PCNM 1. Environmental variables: PC 1- development to shrubland, PC 2- pasture to development, PC 3- emergent wetlands to forests. Spatial variables: PCNM 30- fine spatial scales to PCNM 1- coarse spatial scales. (DOCX) [file pone.0271405.s002.docx]

**S1 Table. Canonical correspondence analysis with avian species.**

|  | Environmental | | Spatial | |
| --- | --- | --- | --- | --- |
| Species | Axis 1 | Axis 2 | Axis 1 | Axis 2 |
| Acadian Flycatcher | 0.558 | 0.383 | -0.625 | 0.223 |
| Alder Flycatcher | -0.064 | -0.213 | -0.101 | 0.160 |
| American Crow | -0.309 | 0.260 | 0.078 | -0.267 |
| American Goldfinch | 0.292 | -0.388 | -0.330 | -0.401 |
| American Redstart | 0.253 | 0.046 | -0.241 | 0.168 |
| American Robin | -0.175 | -0.134 | 0.098 | -0.115 |
| Ash-throated Flycatcher | -0.135 | -0.092 | 0.051 | 0.284 |
| Audubon's Oriole | 0.212 | 0.490 | -0.679 | 0.140 |
| Baird's Sparrow | -0.644 | -0.539 | 0.236 | 0.838 |
| Baltimore Oriole | 0.321 | -0.041 | -0.285 | 0.344 |
| Bank Swallow | 0.120 | -0.047 | -0.303 | 0.034 |
| Barn Swallow | -0.066 | 0.115 | 0.196 | -0.323 |
| Bay-breasted Warbler | 0.709 | 0.137 | -0.531 | 0.218 |
| Bell's Vireo | -0.218 | -0.496 | -0.166 | 0.393 |
| Bewick's Wren | -0.071 | -0.203 | 0.062 | 0.062 |
| Black Phoebe | -0.433 | -0.361 | -0.323 | 0.858 |
| Black-and-white Warbler | -0.011 | -0.111 | -0.237 | 0.070 |
| Blackburnian Warbler | 0.323 | 0.123 | -0.312 | 0.123 |
| Black-capped Vireo | -0.456 | -0.525 | -0.231 | -0.024 |
| Black-chinned Sparrow | -0.125 | -0.170 | -0.196 | 0.172 |
| Black-crested Titmouse | -0.188 | 0.054 | -0.092 | 0.266 |
| Black-headed Grosbeak | 0.165 | -0.791 | -0.027 | 0.458 |
| Blackpoll Warbler | 1.517 | 0.763 | 0.285 | 0.003 |
| Black-tailed Gnatcatcher | -0.427 | -0.715 | 0.692 | 0.964 |
| Black-throated Blue Warbler | 1.868 | 0.138 | -0.622 | -1.387 |
| Black-throated Green Warbler | 0.504 | 0.189 | -0.280 | 0.077 |
| Black-throated Sparrow | -0.267 | -0.668 | 0.150 | 0.594 |
| Blue Grosbeak | -0.024 | 0.055 | -0.014 | -0.035 |
| Blue Jay | -0.292 | 0.083 | 0.185 | -0.030 |
| Blue-gray Gnatcatcher | -0.073 | -0.136 | -0.291 | 0.065 |
| Blue-headed Vireo | 0.342 | -0.368 | -0.135 | 0.235 |
| Blue-winged Warbler | 0.793 | -0.098 | -0.461 | -0.428 |
| Boat-tailed Grackle | 1.452 | 0.603 | 0.102 | -0.678 |
| Botteri's Sparrow | 2.879 | 0.391 | -0.871 | 0.606 |
| Brewer's Blackbird | 0.034 | -0.202 | -0.031 | 0.244 |
| Brewer's Sparrow | -0.578 | -0.873 | 0.605 | 0.705 |
| Bronzed Cowbird | 0.265 | -0.038 | -0.125 | 0.145 |
| Brown Creeper | 0.484 | -0.630 | -0.410 | -0.436 |
| Brown Thrasher | 0.569 | 0.150 | 0.279 | 0.025 |
| Brown-crested Flycatcher | 0.424 | 0.185 | -0.124 | 0.618 |
| Brown-headed Cowbird | -0.087 | 0.156 | 0.186 | -0.124 |
| Bullock's Oriole | 0.040 | -0.624 | 0.115 | -0.069 |
| Cactus Wren | 0.107 | -0.319 | 0.439 | 0.660 |
| Canada Warbler | 0.233 | -0.037 | -0.501 | 0.378 |
| Canyon Towhee | -0.268 | -0.288 | -0.068 | 0.315 |
| Canyon Wren | -0.016 | -0.431 | -0.275 | -0.013 |
| Carolina Chickadee | -0.313 | 0.288 | -0.063 | -0.213 |
| Carolina Wren | -0.267 | 0.188 | 0.070 | -0.095 |
| Cassin's Finch | 0.061 | -0.570 | -1.175 | 0.832 |
| Cassin's Kingbird | -0.585 | -0.755 | 0.632 | 0.754 |
| Cassin's Sparrow | 0.143 | -0.237 | -0.136 | 0.161 |
| Cave Swallow | -0.071 | 0.111 | -0.050 | 0.389 |
| Cerulean Warbler | 1.600 | 0.758 | 0.084 | -0.020 |
| Chestnut-collared Longspur | 0.907 | -0.709 | -0.660 | 0.211 |
| Chestnut-sided Warbler | 0.440 | -0.046 | -0.034 | 0.211 |
| Chihuahuan Raven | -0.061 | -0.279 | 0.194 | 0.792 |
| Chipping Sparrow | -0.004 | -0.007 | -0.094 | 0.218 |
| Clay-colored Sparrow | 0.226 | -0.641 | 0.082 | -0.253 |
| Cliff Swallow | -0.212 | 0.126 | 0.155 | -0.201 |
| Common Grackle | -0.148 | 0.124 | 0.125 | -0.556 |
| Common Ground-Dove | 0.288 | -0.430 | 1.471 | 0.589 |
| Common Raven | -0.069 | -0.136 | -0.117 | 0.315 |
| Common Yellowthroat | 0.240 | -0.171 | -0.202 | -0.041 |
| Cordilleran Flycatcher | -0.578 | -0.873 | 0.605 | 0.705 |
| Couch's Kingbird | 0.224 | 0.227 | -0.563 | 0.869 |
| Curve-billed Thrasher | 0.403 | -0.180 | 0.008 | 0.185 |
| Dark-eyed Junco | 0.329 | 0.222 | -0.631 | 0.130 |
| Dusky Flycatcher | -0.578 | -0.873 | 0.605 | 0.705 |
| Eastern Bluebird | -0.232 | 0.152 | -0.078 | -0.152 |
| Eastern Kingbird | 0.176 | 0.149 | -0.167 | -0.019 |
| Eastern Meadowlark | -0.106 | 0.417 | 0.017 | -0.315 |
| Eastern Phoebe | -0.115 | -0.150 | -0.029 | 0.051 |
| Eastern Towhee | 0.907 | -0.709 | -0.660 | 0.211 |
| Eastern Wood-Pewee | 0.022 | 0.450 | -0.094 | -0.305 |
| Eurasian Collared-Dove | -0.109 | 0.105 | 0.550 | -0.079 |
| European Starling | -0.153 | 0.146 | 0.075 | -0.168 |
| Field Sparrow | -0.161 | -0.135 | -0.250 | -0.096 |
| Fish Crow | -1.228 | -0.963 | -0.198 | -0.482 |
| Fox Sparrow | 0.341 | -0.548 | 0.184 | 0.198 |
| Golden-cheeked Warbler | -0.489 | -0.176 | -0.656 | 0.454 |
| Golden-crowned Kinglet | 0.763 | 0.357 | -0.396 | -0.720 |
| Golden-winged Warbler | 1.319 | 0.496 | 0.087 | -0.095 |
| Grace's Warbler | 0.061 | -0.570 | -1.175 | 0.832 |
| Grasshopper Sparrow | 0.134 | 0.096 | -0.383 | -0.679 |
| Gray Catbird | 0.292 | -0.299 | -0.180 | -0.217 |
| Gray Flycatcher | 1.868 | 0.138 | -0.622 | -1.387 |
| Gray Vireo | 1.868 | 0.138 | -0.622 | -1.387 |
| Gray-cheeked Thrush | 1.369 | 0.269 | -0.164 | 0.057 |
| Great Crested Flycatcher | -0.058 | -0.097 | -0.188 | 0.022 |
| Great Kiskadee | 0.424 | 0.266 | -0.629 | 0.871 |
| Great-tailed Grackle | -0.050 | -0.085 | 0.112 | -0.134 |
| Green Jay | -0.278 | 0.311 | 0.102 | 0.780 |
| Green-tailed Towhee | -0.058 | -0.308 | 0.039 | 0.617 |
| Harris's Sparrow | -0.496 | 0.080 | -0.202 | 0.096 |
| Hepatic Tanager | 0.645 | -0.368 | -0.008 | -0.341 |
| Hermit Thrush | 0.177 | -0.525 | -0.196 | -0.182 |
| Hooded Oriole | -0.636 | -0.340 | 0.494 | 1.254 |
| Hooded Warbler | 0.654 | 0.102 | -0.193 | 0.407 |
| Horned Lark | 0.325 | 0.554 | 0.010 | -0.654 |
| House Finch | -0.077 | -0.174 | 0.055 | -0.097 |
| House Sparrow | -0.123 | 0.013 | 0.062 | -0.142 |
| House Wren | -0.393 | -0.301 | -0.711 | 0.161 |
| Hutton's Vireo | -0.032 | -0.628 | -0.418 | -0.167 |
| Inca Dove | -0.056 | 0.403 | 0.594 | 0.300 |
| Indigo Bunting | 0.126 | -0.202 | -0.087 | -0.163 |
| Juniper Titmouse | 0.234 | -0.465 | -0.665 | -0.346 |
| Kentucky Warbler | 0.484 | -0.640 | -0.918 | 0.521 |
| Lapland Longspur | 0.907 | -0.709 | -0.660 | 0.211 |
| Lark Sparrow | -0.154 | 0.085 | 0.004 | -0.014 |
| Lazuli Bunting | 0.318 | -1.149 | 0.279 | -1.512 |
| Least Flycatcher | -0.030 | -0.183 | -0.266 | 0.186 |
| LeConte's Sparrow | 0.671 | -1.652 | -0.024 | -0.234 |
| Lesser Goldfinch | 0.002 | -0.144 | -0.290 | 0.011 |
| Lincoln's Sparrow | 0.537 | -0.261 | -0.029 | -0.300 |
| Loggerhead Shrike | 0.197 | 0.122 | -0.055 | -0.271 |
| Long-billed Thrasher | 0.899 | 0.315 | -0.608 | 0.150 |
| Louisiana Waterthrush | -0.207 | -0.322 | 0.096 | 0.019 |
| MacGillivray's Warbler | 0.449 | -0.525 | 0.117 | -0.656 |
| Magnolia Warbler | 0.426 | 0.038 | -0.383 | 0.147 |
| Marsh Wren | 1.061 | 0.227 | -0.286 | 0.062 |
| McCown's Longspur | 0.907 | -0.709 | -0.660 | 0.211 |
| Mexican Jay | -0.340 | -0.826 | 0.597 | 0.624 |
| Monk Parakeet | 0.244 | -1.205 | -0.031 | -0.322 |
| Mountain Chickadee | 0.320 | -0.413 | -0.410 | -0.935 |
| Mourning Dove | -0.203 | 0.170 | 0.527 | -0.072 |
| Mourning Warbler | 0.138 | -0.183 | -0.295 | 0.390 |
| Nashville Warbler | 0.009 | -0.214 | -0.068 | 0.375 |
| Nelson's Sparrow | 1.868 | 0.138 | -0.622 | -1.387 |
| Northern Cardinal | -0.210 | 0.269 | 0.190 | -0.096 |
| Northern Mockingbird | -0.197 | 0.165 | 0.153 | -0.117 |
| Northern Parula | -0.041 | 0.124 | -0.627 | 0.235 |
| Northern Rough-winged Swallow | 0.004 | 0.091 | -0.176 | -0.075 |
| Northern Waterthrush | 0.141 | 0.157 | -0.080 | 0.130 |
| Olive Sparrow | 0.378 | 0.337 | -0.236 | 0.716 |
| Olive-sided Flycatcher | -0.027 | 0.222 | -0.291 | 0.064 |
| Orange-crowned Warbler | 0.333 | -0.300 | -0.186 | -0.128 |
| Orchard Oriole | -0.110 | 0.016 | -0.029 | -0.139 |
| Painted Bunting | -0.178 | 0.106 | 0.048 | -0.061 |
| Painted Redstart | -1.203 | -0.344 | 0.169 | 1.536 |
| Palm Warbler | -0.160 | -0.836 | -0.429 | -0.135 |
| Philadelphia Vireo | 0.598 | 0.051 | -0.088 | 0.089 |
| Pine Siskin | -0.317 | 0.180 | -0.485 | 0.136 |
| Pine Warbler | -1.553 | 1.161 | -2.039 | 0.766 |
| Prairie Warbler | 1.868 | 0.138 | -0.622 | -1.387 |
| Prothonotary Warbler | -0.029 | 0.012 | 0.053 | 0.233 |
| Purple Finch | 0.521 | 0.281 | -1.374 | -0.238 |
| Purple Martin | -0.022 | 0.117 | 0.166 | -0.144 |
| Pygmy Nuthatch | 0.061 | -0.570 | -1.175 | 0.832 |
| Pyrrhuloxia Cardinal | -0.362 | -0.446 | 0.875 | 1.564 |
| Red-breasted Nuthatch | 0.108 | -0.606 | -0.533 | 0.627 |
| Red-eyed Vireo | 0.081 | -0.104 | -0.385 | 0.032 |
| Red-winged Blackbird | -0.145 | 0.099 | 0.047 | -0.235 |
| Rock Pigeon | -0.073 | 0.029 | 0.436 | 0.019 |
| Rock Wren | -0.421 | -0.772 | 0.105 | 0.838 |
| Rose-breasted Grosbeak | 0.387 | 0.021 | -0.025 | -0.072 |
| Ruby-crowned Kinglet | 0.263 | -0.078 | -0.414 | -0.055 |
| Rufous-crowned Sparrow | -0.372 | -0.438 | -0.465 | -0.118 |
| Savannah Sparrow | 0.021 | -0.071 | -0.130 | -0.125 |
| Say's Phoebe | -0.212 | -0.743 | 0.309 | 0.618 |
| Scarlet Tanager | 0.562 | 0.034 | -0.036 | -0.028 |
| Scissor-tailed Flycatcher | -0.134 | 0.085 | 0.125 | -0.222 |
| Scott's Oriole | -0.336 | -0.751 | -0.023 | 0.470 |
| Seaside Sparrow | 1.243 | 0.836 | 0.464 | -0.323 |
| Sedge Wren | 0.671 | -0.159 | -0.310 | -0.588 |
| Song Sparrow | 0.549 | -0.241 | -0.483 | -0.304 |
| Spotted Towhee | 0.156 | -0.585 | -0.452 | 0.073 |
| Summer Tanager | -0.186 | 0.278 | -0.140 | 0.468 |
| Swainson's Thrush | 0.237 | 0.258 | -0.257 | -0.025 |
| Swainson's Warbler | 1.868 | 0.138 | -0.622 | -1.387 |
| Swamp Sparrow | 0.343 | -0.300 | -0.057 | -0.074 |
| Tennessee Warbler | 0.483 | 0.252 | -0.103 | -0.032 |
| Tree Swallow | 0.243 | -0.361 | -0.351 | -0.323 |
| Tropical Kingbird | 0.526 | 0.288 | -0.569 | 1.486 |
| Tufted Titmouse | -0.301 | 0.114 | -0.065 | -0.359 |
| Varied Bunting | -0.578 | -0.873 | 0.605 | 0.705 |
| Verdin Auripare | -0.362 | -0.446 | 0.875 | 1.564 |
| Vermilion Flycatcher | -0.426 | -0.474 | -0.044 | 0.828 |
| Vesper Sparrow | 0.165 | -0.482 | -0.382 | 0.096 |
| Violet-green Swallow | 0.038 | 0.870 | -0.477 | 0.699 |
| Warbling Vireo | 0.199 | 0.268 | -0.408 | 0.242 |
| Western Bluebird | 0.964 | -0.216 | -0.898 | -0.278 |
| Western Kingbird | -0.023 | -0.068 | 0.212 | -0.139 |
| Western Meadowlark | -0.124 | -0.394 | -0.409 | -0.444 |
| Western Tanager | 0.003 | 0.048 | 0.087 | 0.903 |
| Western Wood-Pewee | -0.298 | -0.264 | -0.011 | 0.265 |
| White-breasted Nuthatch | 0.322 | -0.049 | -0.209 | -0.102 |
| White-crowned Sparrow | 0.374 | -0.156 | -0.187 | -0.167 |
| White-eyed Vireo | -0.159 | 0.233 | 0.074 | 0.050 |
| White-throated Sparrow | 0.582 | -0.578 | -0.579 | 0.107 |
| White-tipped Dove | 0.487 | -0.502 | 1.537 | 0.838 |
| White-winged Dove | 0.004 | -0.075 | 0.536 | 0.183 |
| Willow Flycatcher | 0.043 | -0.265 | -0.118 | 0.150 |
| Wilson's Warbler | 0.095 | -0.437 | -0.279 | 0.066 |
| Winter Wren | -0.113 | -1.385 | 0.704 | 0.210 |
| Wood Thrush | 1.099 | 0.461 | -0.192 | 0.024 |
| Woodhouse's Scrub-Jay | -0.243 | -0.187 | -0.430 | 0.150 |
| Worm-eating Warbler | 1.868 | 0.138 | -0.622 | -1.387 |
| Yellow Warbler | 0.001 | 0.061 | -0.244 | -0.099 |
| Yellow-bellied Flycatcher | -0.068 | 0.185 | -0.545 | 0.024 |
| Yellow-breasted Chat | -0.072 | 0.107 | 0.189 | 0.440 |
| Yellow-green Vireo | 1.868 | 0.138 | -0.622 | -1.387 |
| Yellow-headed Blackbird | 0.071 | -0.090 | -0.243 | -0.200 |
| Yellow-rumped Warbler | 0.204 | -0.178 | -0.362 | -0.041 |
| Yellow-throated Vireo | 0.187 | 0.477 | -0.777 | 0.404 |
| Yellow-throated Warbler | 0.945 | -0.381 | -0.819 | -0.115 |
