# Supplementary material for: Taxonomic and functional components of avian metacommunity structure along an urban gradient
Source: PLoS One. 2022 Aug 9;17(8):e0271405. doi: 10.1371/journal.pone.0271405 (PMC9362948; doi:10.1371/journal.pone.0271405)
Supplement: S2 Table — Statistics from variation partitioning with correspondence analysis to examine the relationship between on avian taxonomy and functional traits and independent variables environmental and spatial variables. Environmental variables represent land-cover and climate data, and spatial variables represent coarse to fine spatial scales. For functional traits, axis 1 was the only significant axis for both environmental and spatial variables. Explained variation is cumulative variation for each axes. (DOCX) [file pone.0271405.s003.docx]

**S2 Table. Statistics from variation partitioning.**

|  |  | Environmental | | |  | Spatial | | |
| --- | --- | --- | --- | --- | --- | --- | --- | --- |
|  |  | Axis 1 | Axis 2 | Axis 3 |  | Axis 1 | Axis 2 | Axis 3 |
| Taxonomy | Eigenvalues | 0.09 | 0.07 | 0.05 | Eigenvalues | 0.10 | 0.09 | 0.06 |
|  | Explained variation | 3.33 | 5.81 | 7.52 | Explained variation | 3.41 | 6.49 | 8.49 |
|  | PC 1 | 0.19 | -0.24 | 0.74 | PCNM 1 | 0.37 | -0.33 | -0.34 |
|  | PC 2 | 0.20 | 0.76 | -0.18 | PCNM 4 | 0.23 | 0.50 | -0.35 |
|  | PC 3 | 0.82 | 0.09 | -0.09 | PCNM 9 | -0.10 | 0.59 | 0.02 |
|  |  |  |  |  | PCNM 14 | -0.38 | -0.14 | -0.48 |
|  |  |  |  |  | PCNM 30 | -0.30 | -0.02 | -0.02 |
|  | Cases | 79 | | |  | 79 | | |
|  | Response Variables | 205 | | |  | 205 | | |
|  | Explanatory | 3 | | |  | 5 | | |
|  | Covariates | 5 | | |  | 3 | | |
|  |  | Axis 1 | Axis 2 | Axis 3 |  | Axis 1 | Axis 2 | Axis 3 |
| Functional Traits | Eigenvalues | 0.15 |  |  | Eigenvalues | 0.23 |  |  |
|  | Explained variation | 20.82 |  |  | Explained variation | 29.43 |  |  |
|  | PC 1 | 0.27 |  |  | PCNM 1 | 0.34 |  |  |
|  | PC 2 | -0.12 |  |  | PCNM 4 | 0.18 |  |  |
|  | PC 3 | 0.40 |  |  | PCNM 9 | -0.14 |  |  |
|  |  |  |  |  | PCNM 14 | -0.25 |  |  |
|  |  |  |  |  | PCNM 30 | -0.30 |  |  |
|  | Cases | 79 | | |  | 79 | | |
|  | Response Variables | 8 | | |  | 8 | | |
|  | Explanatory | 3 | | |  | 5 | | |
|  | Covariates | 5 | | |  | 3 | | |
