# Supplementary material for: Taxonomic and functional components of avian metacommunity structure along an urban gradient
Source: PLoS One. 2022 Aug 9;17(8):e0271405. doi: 10.1371/journal.pone.0271405 (PMC9362948; doi:10.1371/journal.pone.0271405)
Supplement: Attachment — Submitted filename: PONE-D-21-38671_LFB.pdf [file pone.0271405.s004.pdf]

Manuscript
Click here to access/download;Manuscript;Metacommuity Paper _PO.pdf

# Taxonomic and functional components of avian metacommunity structure

Erin E. Stukenholtz[1*¶] and Richard D. Stevens[1,2¶]

[1] Department of Natural Resources Management, Texas Tech University, Lubbock, TX, USA

[2] Natural Science Research Laboratory of the Museum of Texas Tech University, Lubbock, TX, USA

* Corresponding author

E-mail: erin.stukenholtz@ttu.edu

[¶]These authors contributed equally to the manuscript.

ORCID number
Erin Stukenholtz: 0000-0002-8730-8897
Richard Stevens: 0000-0002-9821-0633

[a#]Department of Natural Resources Management, Goddard Building, Texas Tech University, Box 42125, Lubbock TX 79409

# Abstract

Identifying biological processes that structure natural communities has long been of interest to ecologists. Structure may be determined by a variety of processes including differential responses of species to environmental characteristics, regional-level spatial influences such as dispersal, or stochasticity generated from ecological drift. Few studies have used the metacommunity paradigm to investigate avian community composition, yet such a theoretical construct may provide insights into species turnover even in more unnatural settings such as rural/urban gradients. We measured influence of spatial and environmental characteristics on two different aspects of avian community structure across a gradient of urbanization: 1) taxonomic composition and 2) functional richness of diet, foraging strategies, nesting locations and morphology. We also measured the relationship between species traits and environmental variables with an RLQ-fourth corner analysis. Together environmental and spatial processes significantly influenced taxonomic structure and functional richness, but spatial variables accounted for more variation than environmental variables. The average area of urbanization among the communities was 31.04 $km^2$ ± 12.26. Increasing urbanization was positively related to number of canopy foragers, while emergent wetlands were negatively related to species with frugivorous diets or those that nested on cliffs/buildings. Intermediate and fine scale spatial variation was positively related to birds with insectivorous diets but were negatively related to body mass and wingspan. Spatial and environmental factors played an important role in taxonomic and functional structure of the entire metacommunity. This study highlights the importance of studying multiple aspects of biodiversity such as taxonomic and functional dimensions, especially when examining effects of urbanization that ultimately results from complimentary spatial and environmental processes.

# Introduction

Community ecology aims to understand the primary mechanisms influencing species abundance and composition at the local level [1]. Metacommunity theory differentiates local and regional processes that influence community structure [2] and can be simplified into four frameworks: neutral theory, patch-dynamics, species-sorting and mass-effects [2]. Initiated by Hubbell [3], neutral theory assumes differences among species regarding their niches are nonexistent or at least unimportant to community structure and focuses on how randomly fluctuating demographic processes and dispersal limitation influences diversity of local communities [2]. The patch-dynamics framework focuses on how tradeoffs, for example in competitive and dispersal abilities, influence temporal dynamics of communities in environmentally homogenous landscapes [2]. The species-sorting framework focuses on species response to a heterogeneous landscape, whereby patterns of presence and absence of species reflect selection for suitable habitats [2,4]. Building upon the metapopulation framework of sources and sinks, the mass-effects framework focuses on how high rates of dispersal of individuals of multiple species into less suitable habitats facilitates coexistence at the local level [2,4].

Environmental filtering can strongly influence species coexistence [4]. However, environmental characteristics often explain no more than 50% of the variation in taxonomic diversity at the local level [4]. While spatial processes or stochasticity often strongly characterize taxonomic composition of communities [4], environmental filtering can have strong influences on related functional characteristics [4-6]. Functional traits are important characteristics that are relevant to performance and are expected to have a strong association with environmental gradients [4]. By examining a variety of functional traits within a large species pool, it may be possible to gain a deeper understanding of regional species distribution and abundance [4].

Combining functional characteristics with metacommunity models also has the potential to provide understanding of effects of anthropogenic modifications on the contemporary biota [4]. Avian community composition has been frequently examined as a response to urban gradients at local scales [7,8]. Nonetheless, only a few studies have used a metacommunity framework to investigate relative contributions of local environmental and regional spatial processes on avian community composition. Local (e.g., resources, patch quality or species interactions) and regional (e.g., connectivity) processes are hypothesized to influence urban community composition [7, 9]. This suggests that environmental filtering, habitat selection or dispersal may affect urban community structure [7, 9] to an equal or greater extent than natural communities. Furthermore, metacommunity theory can be applied to gain insights into species turnover from rural to urban settings, as well as the traits that are related to environmental filtering [7, 10].

To estimate metacommunity structure, we examined taxonomic (species identity) and functional characteristics (morphology, diet, foraging strategy, nesting location, and native/invasive status) of birds distributed throughout Texas. We examined trait-environmental relationship with RLQ and fourth-corner analyses [11-13]. Both analyses are dependent on three matrices, L (species x site matrix), R (environment x site), and Q (species x trait matrix), but provide different perspectives on the structure of communities [13]. While RLQ analyses examine associations among traits and environmental characteristics from a multivariate perspective [13]. Fourth-corner analysis examines species pairwise comparisons of traits and environmental variables [13]. Because both approaches are complimentary, Dray et al. proposed combining both approaches into one [13].

Increasing urbanization can adversely affect species and can cause strong habitat filtering [9, 14]. In addition, predation on nest sites is often higher in urban areas compared to natural environments [15]. Species with nests close to or on the ground can be more impacted by predation within urban environments [15-17]. Furthermore, there is unequal access to food resources for different dietary groups. Insect populations are lower in cities due to increased use of insecticides [18]. However, increases in seed dispensed by humans cause urban areas to be rich in resources for granivorous bird species [18]. Urbanization increases local extinctions of species and populations [19]. For all these reasons, urban communities tend to be primarily composed of introduced invasive species or highly adaptable native species [20]. Due to the multiple factors mentioned above, we predict that: 1) birds that are granivorous, nest off the ground, or are invasive will be more associated with urban environments, 2) birds that are native, insectivorous, and ground nesters will be more associated with natural environments, and 3) species richness will be lower in areas of high urbanization and will increase in more natural or rural areas.

# Materials and Methods

## Study Area

This study focuses on avian communities in Texas, the state with the greatest number of recorded bird species (647) within the United States [21]. Texas is highly urbanized. Houston (2,303,482), San Antonio (1,492,510), and Dallas (1,317,929), represent metroplexes with three of the ten highest human populations in the United States. Besides being highly urbanized in parts of the state, Texas is composed of multiple ecoregions. Eastern Texas has a gradient of pine forests to prairies with wetlands in the south, while central Texas has a gradient of cross timbers to open grasslands. Continuing westward there is an increase in mesquite, hill country, canyons

and deserts. Precipitation increases from west to east, and temperature increases from north to south [21].

## Bird Data – L matrix

We collected data from the Breeding Bird Survey (https://www.pwrc.usgs.gov/bbs/results/) and eBird (http://ebird.org/ebird/data/download) from 1 May to 31 August 2013 to 2017. The Breeding Bird Survey was developed by the US Geological Survey's (USGS) Patuxent Wildlife Research Center and Environment Canada's Canadian Wildlife Service to monitor North American bird populations. The eBird database is a citizen science project whose data has demonstrated to be effective for description of patterns of diversity at multiple spatial scales and to be comparable to other more standardized data sets [23]. To limit the inherent biases within eBird data, we followed methods developed by Callaghan et al. and Sullivan et al. [24-26]. We removed the following kinds of checklists: (1) those that did not report all observed species, (2) were duplicates from multiple observers who participated in the same sampling event, or (3) situations where the observer traveled greater than 5km or covered more than 500 ha. We kept checklists (4) that had a duration between 5 to 240 minutes and (5) followed traveling, random or stationary protocols. We focused on observations of Passeriformes (215 species), Columbiformes (9 species) and Psittaciformes (2 species). We included orders Columbiformes and Psittaciformes to discern how environmental and spatial processes affect invasive species. Observations from avian surveys were plotted in ArcGIS (ESRI, Redlands, California, USA).

We created a grid across Texas with cells of 40 $km^2$. In the center of each square, we created a buffer with 10 km radius and an area of 314 $km^2$. We expect the buffer to be greater than the home range of all the studied species. Home range size is related to body size in animals

[27, 28]. Studies on species home ranges are limited, but many passerines within this study have a home range of less than 9 $km^2$ [29-31]. Some of the larger species within the orders Passeriformes, Columbiformes, and Psittaciformes have home ranges ranging from 0.005 $km^2$ (feral rock pigeon, *Columba livia*) [32] to 325 $km^2$ (common grackle, *Quiscalus quiscula*)[33]. The buffer used here encompassed the site of multiple species home ranges, suggesting that multiple populations are present within each site. Sites were larger when compared to other studies and this increase can increase the probability of species detection [35]. The 20 km distance between each site is greater than the home range of each studied species, which limits the possibility of a single individual being included in more than one site, thereby enhancing independence of data points. We extracted Birding Bird Survey and eBird GPS points that intersected the buffers to make up the communities. To ensure that we limited analyses to those communities that were well sampled, we removed communities with less than 50 individuals or those that did not exhibit an asymptote in species richness based on a rarefaction curve.

## Trait Data — Q matrix

For ecological traits, we collected information on diet and percent foraging strategies from Wilman et al. [35] and morphological measurements and nesting strategies from Oberholser [36, 37] and Ricklefs [38]. We collected data on native status from Oberholser [36, 37], to which we defined invasive species as non-indigenous birds, whose distribution expanded due to human facilitation. Because dietary variation depends on location and season, we identified species dietary guild using the item that was the highest proportion of their diet, as done by Wilman et al. [35]. In cases where morphological measurements for females and males were collected, we averaged means between the sexes. Furthermore, we used presence (1) or absence (0) for nesting strategies (Table 1). Average body size, dietary characteristics (diet,

foraging strategy and bill length) and nest type are related to environmental characteristics of niches [39-41]. Wing lengths of birds are related to energetic costs of flight and facilitate movements across fragmented locations [42].

**Table 1.** Traits gathered for species in Texas avian metacommunities in the summer of 2013 to 2017.

| Traits | Categories | Metrics |
|---|---|---|
| Dietary Guild | Insectivorous | 0, 1 |
| | Scavenger | |
| | Frugivorous | |
| | Nectarivorous | |
| | Granivorous | |
| | Herbivorous | |
| Foraging Strategy | Ground | % |
| | Understory | |
| | Midhigh | |
| | Canopy | |
| | Aerial | |
| Morphometrics | Body Mass | Grams |
| | Wing Length | Millimeters |
| | Bill Length | |
| Nesting Strategy | Ground | 0, 1 |
| | Shrub | |
| | Tree, cavity | |
| | Tree, cup | |
| | Building, cliffs | |
| | Parasitic | |
| Status | Native | 0, 1 |
| | Invasive | |

We characterized trait richness using Rao's quadratic entropy [43-45]. Trait information was not available for all species; therefore, we preformed these analyses on 189 species, 17 fewer than the taxonomic analyses. Many traditional methods examine trait diversity by organizing species into groups, rather than assessing quantitative species characteristics [45]. We

measured functional trait richness for diet, foraging type, nesting locations, bill length, wing length and body mass by using the R package "SYNCSA" [46]. Furthermore, we calculated species richness of invasive species and native species for each community.

## Spatial and Environmental Data – R matrix

We collected land cover data from the 2016 USGS National Land Cover Dataset that had a resolution of 30 $m^2$ [47]. Using ArcGIS, we aggregated the land cover types within each buffer and then expressed each land cover type as a percentage. Moreover, we extracted the averages of precipitation and temperature from May to August of 2013 to 2017 for each site using PRISM that had a resolution of 4 $km^2$. To obtain spatial coordinates, we extracted projected Universal Transverse Mercator coordinates (WGS 84, Universal Transverse Mercator, zone 14) for the center of each site via ArcGIS.

## Statistical Analyses

To examine metacommunity structure, we created derived environmental variables with a principal components analysis (PCA) and derived spatial variables with principal coordinates of neighborhood matrices (PCNM). For environmental variables, we performed a PCA with a correlation matrix to produce a reduced set of important environmental variables that were orthogonal. We used the broken stick method to determine which PCs were significant (Peres-Neto et al. 2003). To construct PCNM's, we followed the protocol of Borcard and Legendre [48] using packages "vegan" [49] and adespatial [50] in R 4.0.3 by: 1) creating a Euclidean distance matrix, 2) computing principal coordinates from a truncated distance matrix with a defined threshold of 4 times the nearest neighbor sampling distance as suggested by Borcard and Legendre [48], 3) tested significance of the global model with a canonical correspondence analysis (CCA) with species occurrences as the dependent matrix and all PCNMs as the

independent matrix and, 4) after a significant global model, we assessed significance with forward selection based on a CCA and retained only eigenvectors with positive eigenvalues that were significant.

Variation partitioning of a constrained ordination, such as a CCA, partitions variation among multiple groups of explanatory variables [51] and allows for examination of unique variation related to a particular explanatory group (i.e., environment) after controlling for shared variation with other explanatory groups (i.e., space) [51]. We examined relationships among spatial factors, environmental factors and species composition with a partial CCA to determine: 1) species composition uniquely related to environmental characteristics based on significant PCs, 2) species composition uniquely related to spatial factors based on significant PCNMs, and 3) species composition related to spatially structured environmental characteristics [52, 53] in Canoco 5 [54]. The dependent matrix was comprised of species presence/absence for 79 communities, and independent matrices were five significant spatial PCNMs and three significant environmental PCs. Since multiple independent variables are being used, we used adjusted coefficient of determination ($R^2$) to estimate effect size. We used Monte Carlo permutation (999 permutations) to determine significance of unique variation accounted for by environmental and spatial variables. Following a similar procedure as for taxonomic composition using a partial constrained analysis, we examined unique relationships of trait richness with environmental and spatial factors with a partial redundancy analysis (RDA) [52, 53] in Canoco 5 [54].

We examined the relationship between species traits and environmental variables with fourth-corner and RLQ analyses: R represented the site by environment matrix, L represented the site by species matrix, and Q represented the species by trait matrix [51]. Matrices Q and R were

coupled using an ordination to create linear combinations that are then linked to species presence/absence in matrix L [55]. The fourth-corner tests the relationship between traits and environmental variables for each species separately, while an RLQ analysis is a multivariate analysis of the three matrices [13] that examines all species simultaneously. Combining both RLQ and fourth-corner analyses, we tested for global significance by examining two permutation models: Model 2 and Model 4. Model 2 permutes sites to determine if the relationship between species and environment was significant [56]. Model 4 permuted species occurrences to examine if the relationship between species and traits was significant [56]. P-values are estimated by comparing the observed value with statistics of the permutation distribution [13]. This method has been suggested by Dray and Legendre to limit type 1 error [56]. RLQ and fourth-corner analyses were performed using the R package "ade4" [57].

# Results

## Bird Data – L matrix

We obtained data on species composition of 79 well-sampled communities. There were 205 species present within communities, with mourning doves (*Zenaida macroura*) being present at the most locations (97% of sites, Fig 1). Most invasive species were common across Texas: Eurasian collared-dove (*Streptopelia decaocto*; 75% of sites), house sparrow (*Passer domesticus*; 62%), European starling (*Sturnus vulgari*; 56%) and rock pigeon (*Columba livia*; 44%). The monk parakeet (*Myiopsitta monachus*) was the invasive species distributed across the fewest sites being present at only 4%.

**Fig 1. Species Commonality in Texas.** The commonality of avian species throughout the 79 communities during the summer of 2013 to 2017.

Correspondence analysis showed that Columbidae was the only family that had clear separation in ecological space compared to other avian families (Fig 2a). Eurasian collared-doves were closely situated to native Columbiformes, while all other invasive species including rock pigeons were closely located with native species in ecological space.

**Fig 2. Relationship between spatial and environmental variables and taxonomy and functional richness.** Canonical correspondence analysis of species composition with environmental (a) and spatial (b) variables, and redundancy analysis of trait richness with environmental (c) and spatial (d) characteristics for bird species in Texas during the summer of 2013 to 201[illegible]

## Spatial and Environmental Data – R matrix

Most common landcover type among the communities was shrubland with an approximate average area of 94.34 $km^2$ ± 24.03. This is followed by pastures (47.38 $km^2$ ± 14.79), grassland (35.37 $km^2$ ± 12.27), croplands (34.76 $km^2$ ± 13.84), and urbanized areas (31.04 $km^2$ ± 12.26). Principal components analysis on the 15 land cover types and average precipitation and temperature yielded 3 significant PC’s (Table 2) that together account for 55.19% of the variation in environmental characteristics. The first PC accounted for 24.38% of the variation among sites regarding environmental variation and was interpreted as a gradient from shrub and grassland to more developed, urban areas (Table 2). The second PC accounted for 17.12% of the variation and encompassed a gradient of high urbanization to pastoral lands. The last significant PC accounted for 13.69% of the variation and was interpreted as a gradient ranging from forested areas to barren and emergent wetlands. Canonical correspondence analysis

using all positive PCNMs, and the community matrix yielded a significant result ($R^2_{adj}$ = 13%, F = 1.2, P < 0.001). Five PCNMs were significant based on forward selection: PCNM 1 ($R^2_{adj}$ = 2.9%, F = 3.4, P = 0.011), PCNM 4 ($R^2_{adj}$ = 3.1%, F = 3.5, P = 0.004), PCNM 9 ($R^2_{adj}$ = 1.6%, F = 2.3, P = 0.019), PCNM 14 ($R^2_{adj}$ = 3.0%, F = 3.4, P = 0.008), PCNM 30 ($R^2_{adj}$ = 1.5%, F = 2.3, P = 0.035).

**Table 2**. Eigenvalues of significant principal components that are derived from land cover types, precipitation, and temperature characteristics for Texas avian metacommunities in the summer of 2013 to 2017. Bold eigenvalues indicate land cover types that assist in the gradient description

| Variables | Principal Components | | |
|---|---|---|---|
| | 1 | 2 | 3 |
| Barren | 0.13 | 0.18 | **0.65** |
| Cropland | **-0.16** | -0.07 | -0.16 |
| Deciduous Forest | 0.21 | 0.24 | **-0.38** |
| Emergent Wetlands | 0.12 | 0.37 | **0.78** |
| Evergreen Forest | 0.07 | 0.13 | -0.30 |
| Grassland | **-0.20** | -0.32 | -0.19 |
| High Development | **0.83** | **-0.45** | 0.08 |
| Low Development | **0.90** | -0.35 | 0.03 |
| Mid Development | **0.87** | **-0.45** | 0.06 |
| Mixed Forest | 0.21 | **0.58** | **-0.41** |
| Open Development | 0.83 | -0.37 | -0.14 |
| Pasture | 0.22 | **0.68** | **-0.31** |
| Precipitation | 0.64 | 0.48 | -0.20 |
| Shrubland | **-0.61** | **-0.44** | 0.18 |
| Temperature | 0.25 | 0.45 | 0.46 |
| Water | 0.22 | 0.29 | **0.64** |
| Woody Wetland | 0.25 | **0.62** | -0.16 |
| % Variation | 24.38 | 17.12 | 13.69 |

# Variation Partitioning

Environmental variables accounted for a significant amount of unique variation in species composition ($R^2_{adj}$ = 3.6%, F = 1.9, P = 0.001, Fig 2a). The first axis of the CCA was most

associated with environmental PC 3 (r = 0.82, df = 77, P < 0.001), a gradient of forest to emergent wetlands. The second axis of the CCA was most associated with environmental PC 2 (r = 0.76, df = 77, P < 0.001) and to a lesser extent with environmental PC 1 (r = -0.24, df = 77, P = 0.031). Most species had a higher association with urbanization and forested regions. Spatial variables accounted for a higher percent of the variation compared to environmental variables (variation explained = 4.7%, F = 1.7, P = 0.001; Fig 2b). The first axis of the CCA was positively correlated with PCNM 1 and PCNM 4 and was negatively associated with PCNM 14 and PCNM 30 (Table 3). The second axis of the CCA was positively correlated with PCNM 4 and PCNM 9, and negatively correlated with PCNM 1 (Table 3). Combined spatial and environmental variables accounted for 10.5% of the variation (F = 2.01, P = 0.001). The first gradient was strongly related to PCNM 4 (r = 0.58, df = 77, P < 0.001) and increasing urbanization (r = -0.55, df = 77, P < 0.001). The second axis exhibited a positive association with emergent wetlands (PC 3, r = 0.59, df = 77, P < 0.001), PCNM 4 (r = 0.42, df = 77, P < 0.001), and PCNM 9 (r = 0.48, df = 77, P < 0.001).

**Table 3.** Correlations of environmental and spatial to axes of a Canonical Correspondence Analysis of species occurrences and Redundancy Analysis of trait richness for birds in Texas during the summer of 2013 to 2017. Degrees of freedom for both analyses were 77. Bold = P-value less than 0.05. Degrees of freedom for all correlations equal 77.

| | | Species Occurrence | | Trait Richness | |
|---|---|---|---|---|---|
| | | Axis 1 | Axis 2 | Axis 1 | Axis 2 |
| Environmental | PC 1 | 0.19 | **-0.24** | **0.27** | 0.11 |
| | PC 2 | 0.20 | **0.76** | -0.12 | -0.06 |
| | PC 3 | **0.82** | 0.09 | **0.40** | -0.07 |
| Spatial | PCNM 1 | **0.37** | **-0.33** | **0.34** | -0.05 |
| | PCNM 4 | **0.23** | **0.51** | 0.18 | -0.05 |
| | PCNM 9 | -0.10 | **0.59** | -0.14 | 0.15 |
| | PCNM 14 | **-0.38** | -0.15 | **-0.25** | -0.11 |
| | PCNM 30 | **-0.30** | -0.02 | **-0.30** | -0.06 |

There was a significant relationship between trait richness and environmental variables ($R^2_{adj}$ = 17%, F = 6.1, P = 0.001; Fig 2c). Environmental PC 1 and 3 were significantly correlated with the first axis of the RDA (Table 3). However, none of the PCs were correlated with the second axis of the RDA. All trait richness variables, except body mass, were positively associated with increasing urbanization (PC 1) and emergent wetlands (PC 3). Spatial variables accounted for more variation than environmental variables ($R^2_{adj}$ = 24.4%; F = 5.8, P = 0.001; Fig 2d). Spatial variables PCNM 14 and PCNM 30 were negatively correlated with the first axis of the RDA, while PCNM 1 was positively correlated with the first axis of the RDA. Spatially structured environmental characteristics accounted for a significant amount of the variation for community composition ($R^2_{adj}$ = 29.43%; F = 6.8, P = 0.001). The first axis of the RDA of spatially structured environmental variables were correlated with PC 1 (r = 0.27, df = 77, P = 0.014), PC 3 (r = 0.35, df = 77, P = 0.001), PCNM 1 (r = -0.25, df = 77, P = 0.030), PCNM 9 (r = 0.24, df = 77, P = 0.032), PCNM 14 (r = 0.29, df = 77, P = 0.010), PCNM 30 (r = 0.22, df = 77, P = 0.048).

## RLQ and Fourth Corner Analyses

There was a significant global relationship between species occurrences and environmental variables (Model 2: Observed value = 0.25, P < 0.001, Fig 3a). Furthermore, the relationship between species occurrences and traits, while preserving the link between species and environmental variables, was also significant (Model 4: Observed value = 0.25, P < 0.001, Fig 3b). This indicated that there was a significant multivariate pattern between traits and environmental variables. Spatial and environmental variables accounted for 86.29% of the variation in trait variables, with the first RLQ axis accounting for 74.93% of the variation.

**Fig 3. RLQ and fourth-corner analysis.** Combined RLQ and fourth-corner analysis of avian communities in Texas during the summer 2013 to 2017. Multivariate axes position of explanatory variables (a) and traits (b). Combining both fourth-corner and RLQ analysis (c), positive (---) and negative (- -) associations among significant (black) and nonsignificant variables (gray) in multivariate spaces (as they are found in a and b). Species organized by families in multivariate space according to the RLQ analysis (d).

Fourth-corner analysis indicated that 8 out of 176 bivariate associations were significant (Fig 3c). Environmental principal component 1 was positively associated with foraging at canopy level (r = 0.09, P = 0.038). Environmental principal component 3 was negatively associated with a frugivorous diet (r = -0.06, P = 0.038) and nesting on cliffs or buildings (r = -0.07, P = 0.036). For spatial variables, PCNM 4 was positively related to nesting in shrubs (r = 0.07, P = 0.036), PCNM 14 and PCNM 30 were positively related to insectivorous diet (r = 0.10, P = 0.038; r = 0.10, P = 0.038), and PCNM 30 was negatively related to body mass (r = -0.070, P = 0.036) and wing length (r = -0.09, P = 0.038).

There were significant associations between RLQ axes, traits and explanatory variables when combining fourth-corner and RLQ approaches (Table 4). The first RLQ axis was negatively correlated with environmental PC 1, environmental PC 3, PCNM 9, PCNM 14 and PCNM 30. For trait variables, RLQ axis 1 was negatively associated with an insectivorous diet, foraging at mid-high and canopy level, and being native. In contrast, the first axis of the RLQ was positively related to granivorous diets, body mass, wing length, nesting in shrubs, and being invasive. Axis 2 was significantly related to PCNM 4 but no other variables. Communities in areas of increased urbanization and emergent wetlands were mostly associated with insectivorous, native species, and species that forage at mid-high to canopy level. Individuals

from the families Hirundinidae, Parulidae, Polioptilidae, Sittidae, Tyrannidae, Troglodytidae, Vireonidae, and Sittidae were positively correlated with increasing urbanization, emergent wetlands, PCNM 14 and PCNM 30 (Fig 3d). However, individuals from Corvidae, Sturnidae, Passeridae, and Columbidae were negatively associated with the gradient.

**Table 4.** RLQ Axes correlations with trait and explanatory variables. Bold = P-value less than 0.05.

| | | | Axis 1 | Axis 2 |
|---|---|---|---|---|
| Traits | Diet | Insectivorous | **-0.15** | -0.02 |
| | | Scavenger | 0.01 | 0.04 |
| | | Frugivorous | 0.06 | 0.04 |
| | | Nectarivorous | 0.02 | -0.03 |
| | | Granivorous | **0.15** | 0.01 |
| | | Plants | 0.04 | 0.01 |
| | Foraging | Ground | 0.12 | -0.01 |
| | | Understory | -0.02 | 0.01 |
| | | Midhigh | **-0.09** | < 0.01 |
| | | Canopy | **-0.13** | 0.01 |
| | | Aerial | 0.01 | -0.01 |
| | Morphometrics | Body Mass | **0.09** | < -0.00 |
| | | Wing Length | **0.14** | -0.01 |
| | | Bill Length | 0.06 | -0.02 |
| | Nesting type | Ground | -0.04 | 0.02 |
| | | Shrub | **0.04** | -0.07 |
| | | Tree Cavity | 0.01 | 0.04 |
| | | Tree Cups | 0.06 | -0.04 |
| | | Building/Cliffsides | 0.01 | 0.04 |
| | | Parasitic | 0.01 | -0.01 |
| | Status | Invasive | **0.10** | 0.01 |
| | | Native | **-0.10** | -0.01 |
| Explanatory | Environmental | PC1 | **-0.09** | 0.05 |
| | | PC 2 | -0.01 | -0.02 |
| | | PC 3 | **-0.01** | -0.06 |
| | Spatial | PCNM 1 | 0.06 | < -0.01 |
| | | PCNM 4 | 0.07 | **-0.07** |
| | | PCNM 9 | **-0.11** | -0.05 |
| | | PCNM 14 | **-0.09** | 0.01 |
| | | PCNM 30 | **-0.11** | 0.01 |

# Discussion

Urbanization, land-use and climate were not primary driving forces of taxonomic and functional metacommunity structure. While significant, spatial and environmental processes accounted for less than 25% of variation in taxonomic characteristics or trait richness. Spatial characteristics accounted for more unique variation than environmental characteristics with respect to taxonomic components and trait diversity. Spatial and environmental variables also accounted for a large portion of the variation in RLQ matrices. However, environmental variables, specifically urbanization, were not as influential in community structure as we originally predicted. Canopy foraging was the only trait significantly related to increased urbanization.

In a meta-analysis of metacommunities conducted by Cottenie [53], the majority of studies demonstrated that environmental factors were the main driving force of community structure, indicating that species-sorting was the most common framework explaining metacommunity structure. The second most prominent structure was a combination of spatial and environmental variables indicating a combination of mass-effects and species-sorting [53]. Of the 158 studies, the four avian metacommunity studies examined exhibited species-sorting or mass-effects [53]. Other avian metacommunity studies have also detailed the importance of environmental variables (species-sorting framework) on community structure [58-62]. Similar to our study, stochasticity was a major characteristic of metacommunity structure in a human-modified landscape in France, but environmental filtering and dispersal were significant contributors [62].

Our study demonstrated that environmental filtering played a significant role in taxonomic and functional components of community structure. The gradient of emergent wetlands to forests played a major role in functional richness and species presence. Mesic conditions within wetlands can contribute to an increase in food and water availability, being related to increased avian richness and abundance compared to uplands [63, 64]. Richness in bill lengths, wing lengths and foraging strategies had the highest association with wetlands and Passerine dietary niches were related to their bill size, locomotion and foraging behavior in passerines [65]. Bill length has been demonstrated to be longer for passerines in wetlands than in species from drier lands [66]. Increased bill length can facilitate greater dietary breadth by increasing ability to reach insects in crevices [65, 67].

There was a negative relationship between occurring in wetlands and having a frugivorous diet or nesting on buildings/cliffs. While longer bills may be more beneficial for birds in wetland areas, wider, well-developed, and sometimes shorter bills provide advantages to frugivores [68]. Shorter bills allow birds to better manipulate fruit [68]. Moreover, birds that nest on buildings or cliff sides tend to occur within cities especially in regions with little topographic relief such as the high plains of Texas. Canyons and mountains are in the western arid regions of Texas and serve as valuable nesting locations for cliff dwelling birds. Buildings within cities can provide pseudo-cliff structures for nesting, which invasive species such as feral pigeons take advantage of when nesting in cities [69].

We predicted that urbanization would influence species composition and functional traits. However, the gradient of shrubland to urbanization was not significantly related to species composition but was correlated with invasive species and nesting richness. Urbanization greatly influences biodiversity via multiple synergistic mechanisms [9]. Although these mechanisms can

impact multiple native species, invasive birds thrive in cities and can outcompete similar native species [70-72]. Adaptations, such as behavioral flexibility [10, 73, 74], ecological generalism [75, 76], certain life history traits such as brood value (how many broods over a lifetime) [76] and human tolerance [74, 77] have all been attributed to success of invasive species in urban environments.

Native species and urbanization had a negative association with RLQ axis 1 (Fig. 5). Urbanized areas are characterized by decreased biodiversity compared to surrounding natural habitat [9]. However, there are many attributes that can increase biodiversity in cities such as higher productivity and resource availability [9]. Increased primary productivity via urban parks can alleviate the negative effects of urbanization [9] or the harsh environment of arid cities [78]. Parks can increase richness by contributing a wide variety of food and nesting sites [79]. Ground nesting birds often are more prevalent in parks, while cavity and tree nesters are often more prevalent in allotment garden (e.g., community garden) [80]. Canopy foragers are often prevalent in parks [81], explaining the positive correlation between urbanization and canopy foragers. While cities are notoriously known for decreasing biodiversity, parks within cities may mitigate this effect [80]. Due to the increase in food, water, and temperature (heat-island effect), birds may be able to thrive in many cities [9, 81]. Therefore, examining characteristics within cities may be key to understanding how urbanization influences species.

Principal coordinates of neighborhood matrices represent eigenvector decompositions of spatial scales and sites [83]. Following Borcard and Legendre classification, PCNMs for this study can be classified into three different spatial groups: broad (PCNM 1, 4 and 9), intermediate (PCNM 14) and fine scale (PCNM 30) [83]. For the multivariate analysis of traits, moderate to fine scale spatial variables were correlated with nesting in shrubs, body mass, wing length and

insectivory. Similar to variation partitioning, correlations are potentially rooted in environmental variables that are related to finer spatial scales [84] or biotic processes (e.g., competition) [83]. My study focused on broad scale landscape variables. However, habitat quality (e.g., food availability) can influence avian distributions [85]. Because of high mobility and larger species having larger home ranges [27, 28], the negative correlation of the fine scale PCNM 30 with body mass and wing length may be related to dispersal ability.

Future studies would benefit from more censuses in the western region of Texas and from considering more environmental variables. Most of the communities come from the eastern region, which is probably due to human population density being greater in the east. Moreover, vegetation complexity and quality are influential to avian distribution and the addition of these variables to the model might improve understanding of metacommunity structure.

Overall, this study provides insight into how spatial and environmental variables play an influential role in avian community structure. Surprisingly, urbanization, or other measured environmental variables, were not the central driving force in metacommunity structure. Spatial variables play a pivotal role in avian communities, whether they be due to dispersal, environmental variables that are spatially structured, or biotic interactions.

# Acknowledgments

We would like to thank Macy Krishnamoorthy and Ty Hopper with reviewing this manuscript.

# References

1. Padial AA, Ceschin F, Declerck SA, De Meester L, Bonecker CC, Lansac-Tôha FA, Rodrigues L, Rodrigues LC, Train S, Velho LFM, Bini LM, Dispersal ability determines the role of environmental, spatial and temporal drivers of metacommunity structure. PLoS One 2014; 9: e111227.

2. Leibold MA, Holyoak M, Mouquet N, Amarasekare P, Chase JM, Hoopes MF, Holt RD, Shurin JB, Law R, Tilman D, Loreau M, Gonzalez A, The metacommunity concept: a framework for multi-scale community ecology. Ecol. Lett. 2004; 7: 601-613.
3. Hubbell SP, The Unified Neutral Theory of Biodiversity and Biogeography. Princeton University Press, Princeton, New Jersey; 2001.
4. Leibold MA, Chase JM, Metacommunity ecology. Vol. 59. Princeton University Press; 2017.
5. Spasojevic MJ, Copeland S, Suding KN, Using functional diversity patterns to explore metacommunity dynamics: a framework for understanding local and regional. Ecography 2014; 37: 939-949.
6. Weinstein BG, Tinoco B, Luis Parra J, Brown LM, McGuire JA, Stiles FG, Graham CH, Taxonomic, phylogenetic, and trait beta diversity in South American hummingbirds. Am. Nat. 2014; 184: 211-224.
7. Johnson PTJ, Hoverman JT, McKenzie VJ, Blaustein AR, Richgels KLD, Urbanization and wetland communities: applying metacommunity theory to understand the local and landscape effects. J. Appl. Ecol. 2013; 50: 34-42.
8. Bellocq M I, Leveau LM, Filloy J, Urbanization and bird communities: Spatial and temporal patterns emerging from southern South America. In: Murgui E, Hedblom M (ed) Ecology and conservation of birds in an urban environment, Spring, Cham, Switzerland; 2017. pp 35-54
9. Shochat, E, Warren, PS, Faeth, SH, McIntyre, NE, Hope, D, From patterns to emerging processes in mechanistic urban ecology. Trends Ecol. Evol 2006; 21: 186-191.
10. Kark S, Iwaniuk A, Schalimtzek A, Banker E, Living in the city: can anyone become an 'urban exploiter'? J. Biogeogr. 2007; 34: 638-651.
11. Dolédec S, Chessel D, ter Braak CJF, Champely S, Matching species traits to environmental variables: a new three-table ordination method. Environ. Ecol. Stat. 1996; 3: 143-166.
12. Legendre P, Galzin R, Harmelin-Vivien ML, Relating behavior to habitat: solutions to the fourth-corner problem. Ecology 1997; 78: 547-562.
13. Dray S, Choler P, Dolédec S, Peres-Neto PR, Thuiller W, Pavoine S, ter Braak CJF, Combining the fourth-corner and the RLQ methods for assessing trait responses to environmental variation. Ecology 2014; 95: 14-21.
14. Swan CM, Pickett S, Szlavecz K, Warren P, Willey K, Biodiversity and community composition in urban ecosystems: coupled human, spatial and metacommunity processes. In: Niemelä J, Breuste JH, Guntenspergen G, McIntyre NE, Elmqvist T, James P (ed.) Urban Ecology: Patterns Processes and Applications, New York City, New York United States, Oxford University Press. 2011; pp. 179–186.
15. Jokimäki J, Huhta E, Artificial nest predation and abundance of birds along an urban gradient. Condor 2000; 4: 838-847.
16. Chace JF, Walsh JJ, Urban effects on native avifauna: a review. Landsc. Urban Plan.2006; 74: 46-49.
17. Croci S, Butet A, Clergeau P, Does urbanization filter birds on the basis of their biological traits? Condor 2008; 110: 223–240
18. Litteral, J, Shochat, E The role of landscape-scale factors in shaping urban bird communities. In Ecology and conservation of birds in urban environments Springer, Cham. 2017; pp. 135-159.
19. McKinney M. L, Urbanization as a major cause of biotic homogenization. Biol. Conserv. 2006; 127: 247-260.

20. Gil D, and Henrik B, Avian urban ecology: behavioural and physiological adaptations. Oxford, United Kingdom, OUP Oxford; 2014.
21. Shackelford CE, Lockwood MW, The Birds of Texas: Occurrence and Seasonal Movements. Texas Parks and Wildlife; 2005.
22. TPWD, Texas Ecoregions. 2020 [January 19, 2021] Available from https://tpwd.texas.gov/education/hunter-education/online-course/wildlife-conservation/texas-ecoregions.
23. Callaghan CT, Gawlik DE, Efficacy of eBird data as an aid in conservation planning and monitoring. J. Field Ornithol. 2015; 86: 298-304.
24. Callaghan CT, Lyons MB, Martin JM, Major RE, Kingsford RT, Assessing the reliability of avian biodiversity measures of urban greenspaces using eBird citizen science data. Avian Conserv. Ecol. 2017; 12:12.
25. Callaghan CT, Major RE, Lyons MB, Martin JM, Wilshire JH, Kingsford RT, Cornwell WK, Using citizen science data to define and track restoration targets in urban areas. J. Appl. Ecol. 2019; 56: 1998-2006.
26. Sullivan BL, Aycrigg JL, Barry JH, Bonney RE, Bruns N, Cooper CB, Damoulas T, Dhondt AA, Dietterich T, Farnsworth A, Fink D, Fitzpatrick JW, Fredericks T, Gerbracht J, Gomes C, Hochachka WM, Illif MJ, Lagoze C, Kelling S, The eBird enterprise: an integrated approach to development and application of citizen science. Biol. Conserv. 2014; 169: 31-40.
27. Lindstedt SL, Miller BJ, Buskirk SW, Home range, time and body size in mammals. Ecology 1986; 67: 413–418.
28. Haskell JP, Ritchie ME, Olff H, Fractal geometry predicts varying body size scaling relationships for mammal and bird home ranges. Nature 2002; 418: 527-530.
29. Schoener TW, Sizes of feeding territories among birds. Ecology 1968; 49:123-141.
30. Wells KMS, Millspaugh JJ, Ryan MR, Hubbard MW, Factors affecting home range size and movements of post-fledging grassland birds. Wilson J. Ornithol. 2008; 120: 120-130.
31. Whitaker DM, Warkentin IC, Spatial ecology of migratory passerines on temperate and boreal forest breeding grounds. Auk 2010; 127: 471-484.
32. Sol D, Senar JC, Urban pigeon populations: stability, home range, and the effect of removing individuals. Can. J. Zool. 1995; 73: 1154-1160.
33. Bray OE, Royall WC Jr, Guarino JL, and Johnson RE, Activities of radio-equipped Common Grackles during fall migration. Wilson J. Ornithol. 1979; 78-87.
34. Thompson WL, Towards reliable bird surveys: accounting for individuals present but not detected. Auk 2002; 119: 18-25.
35. Wilman H, Belmaker J, Simpson J, de la Rosa C, Rivadeneira MM, Jetz W, Elton Traits 1.0: Species-level foraging attributes of the world's birds and mammals: Ecological Archives E095-178. Ecology 2014; 95: 2027-2027.
36. Oberholser HC, The bird life of Texas, volume 1. University of Texas Press, Austin, U.S.A; 1974.
37. Oberholser HC (1974) The bird life of Texas, volume 2. University of Texas Press, Austin, U.S.A.; 1974.
38. Ricklefs RE, Passerine morphology: external measurements of approximately one-quarter of passerines of bird species. Ecology 2017; 98: 1472.
39. Elton C, Animal Ecology. London, United Kingdom; 1927.
40. Van Valen L, Morphological variation and width of ecological niche. Am. Nat. 1965; 99: 370-399.

41. Cody ML, Diamond JM, Ecology and evolution of communities. The Belknap Press of Harvard University Press. Cambridge, Massachusetts and London, England; 1975.
42. Hinsley SA, The cost of multiple patch use by birds. Landsc. Ecol. 2000; 15: 765-775.
43. Rao CR, Diversity and dissimilarity coefficients: a unified approach. Theor. Popul. Biol. 1982; 21: 24-43.
44. Botta-Dukát Z, Rao's quadratic entropy as a measure of functional diversity based on multiple traits. J. Veg. Sci. 2005; 16: 533-540.
45. Kohli BA, Jarzyna MA, Pitfalls of ignoring trait resolution when drawing conclusions about ecological processes. Glob. Ecol. Biogeogr. 2021; 30: 1139-1152.
46. Debastiani V, Pillar V, SYNCSA - R tool for analysis of metacommunities based on functional traits and phylogeny of the community components. Bioinformatics 2012; 28: 2067-2068.
47. Homer CG, Dewitz JA, Jin S, Xian G, Costello C, Danielson P, Gass L, Funk M, Wickham J, Stehman S, Auch RF, Riitters KH, Conterminous United States land cover change patterns 2001–2016 from the 2016 National Land Cover Database: ISPRS J. Photogramm. v. 2016; 162: 184–199.
48. Borcard D, Legendre P, All-scale spatial analysis of ecological data by means of principal coordinates of neighbour matrices. Ecol. Modell. 2002; 153: 51-68.
49. Oksanen J, Guillaume Blanchet F, Friendly M, Kindt R, Legendre P, McGlinn D, Minchin PR, O'Hara RB, Simpson GL, Solymos P, Stevens MHM, Szoecs E, Wagner H, Package "vegan": Community Ecology Package 2020; 10.631-637: 719.
50. Dray S, Blanchet G, Borcard D, Guenard G, Jombart T, Larocque G, Legendre P, Madi N, Wagner HH, "Package ‘adespatial’." R Package 2018; 3-8.
51. Legendre P, Legendre L, Canonical analysis. In: Numerical Ecology Volume 3. Elsevier. Langford Lane, Kidlington, Oxford, United Kingdom. 2012; pp. 625-710.
52. Borcard D, Legendre P, Drapeau P, Partialling out the Spatial Component of Ecological Variation. Ecology 1992; 73: 1045–55.
53. Cottenie K, Integrating environmental and spatial processes in ecological community dynamics. Ecol. 2005; 8: 1175-1182.
54. ter Braak CJF, Šmilauer P, Canoco reference manual and user's guide: software for ordination, version 5.0. Microcomputer Power, Ithaca, New York; 2012.
55. Dray S, Chessel D, Thioulouse J, Co-interia analysis and the link of ecological data tables. ESA 2003; 84: 3078-3089.
56. Dray S, Legendre P, Testing the species traits–environment relationships: the fourth-corner problem revisited. Ecology 2008; 89: 3400– 3412.
57. Dray S, Dufour AB, The ade4 package: implementing the duality diagram for ecologists. J. Stat. Softw. 2007; 22: 1-20.
58. Meynard CN, Quinn JF, Bird metacommunities in temperate South American forest: vegetation structure, area, and climate effects. Ecology 2008; 89: 981-990.
59. Presley SJ, Cisneros LM, Patterson BD, Willig MR, Vertebrate metacommunity structure along an extensive elevational gradient in the tropics: a comparison of bats, rodents and birds. Glob. Ecol. Biogeogr. 2012; 21: 968-976.
60. Bonthoux S, Balent G, Bird metacommunity processes remain constant after 25 years of landscape changes. Ecol. Complex. 2015; 21: 39-43.
61. Henry DA, Ament JM, Cumming GS, Exploring the environmental drivers of waterfowl movement in arid landscapes using first-passage time analysis. Mov. Ecol. 2016; 4: 1-18.

62. Henckel L, Meynard CN, Devictor V, Mouquet N, Bretagnolle V, On the relative importance of space and environment in farmland bird community assembly. PloS One 2019; 14: e0213360.
63. McKinney RA, Paton PWC, Breeding birds associated with seasonal pools in the northeastern United States. J. Field Ornithol. 2009; 80: 380-386.
64. McKinney, RA, Raposa, KB, Cournoyer, RM, Wetlands as habitat in urbanizing landscapes: patterns of bird abundance and occupancy. Landscape and Urban Planning 2011; 100: 144-152.
65. Brandl R, Kristin A, Leisler B, Dietary niche breadth in a local community of passerine birds: an analysis using phylogenetic contrasts. Oecologia 1994; 98: 109-116.
66. Luther D, Greenberg R, The island syndrome in coastal wetland ecosystems: convergent evolution of large bills in mangrove passerines. Auk 2011; 128: 201-204.
67. Post W, Greenlaw JS, Seaside Sparrow (*Ammodramus maritimus*). In: Poole A, Stettenheim P, Gill F (ed.) The Birds of North America, no. 127. Academy of Natural Sciences, Philadelphia, and American Ornithologists' Union, Washington, D.C. 1994; pp 1-27.
68. Moermond TC, Denslow JS, Neotropical avian frugivores: patterns of behavior, morphology, and nutrition, with consequences for fruit selection. Ornithol. Monogr. 1985; 865-897.
69. Johnston RF, Janiga M, Feral pigeons (Vol. 4). Oxford University Press; 1995.
70. Bennett WA, Scale of investigation and the detection of competition: an example from the house sparrow and house finch introductions in North America. Am. Nat. 1990; 135: 725–747.
71. Macías Garcia C, Suárez-Rodríguez M, López-Rull I, Becoming citizens: avian adaptations to urban life. In: Murgui E, Hedblom M (ed.) Ecology and conservation of birds in an urban environment Cham, Switzerland. Springer. 2017; pp. 91-112.
72. Sol D, Gonzalez-Lagos C, Lapiedra O, Diaz M, Why are exotic birds so successful in urbanized environments In: Murgui E, Hedblom M (ed.) Ecology and conservation of birds in an urban environment, Cham, Switzerland, Springer. 2017; pp. 75-90.
73. Møller AP, Successful city dwellers: a comparative study of the ecological characteristics of urban birds in the western Palearctic. Oecologia 2009; 159:849–858
74. Carrete M, Tella JL, Inter-individual variability in fear of humans and relative brain size of the species are related to contemporary urban invasion in birds. PLoS One 2011; 6:e18859. doi:10.1371/journal.pone.0018859
75. Evans K, Chamberlain D, Hatchwell BJ, Gregory RD, Gaston KJ, What makes an urban bird? Glob. Change Biol. 2011; 17: 32-44.
76. Sol D, González-Lagos C, Moreira D, Maspons J, Lapiedra O, Urbanisation tolerance and the loss of avian diversity. Ecol. Lett. 2014; 17: 942-950.
77. Møller AP, Flight distance of urban birds, predation, and selection for urban life. Behav. Ecol. Sociobiol. 2008; 63: 63-75.
78. Mills GS, Dunning JB Jr., Bates JM, Effects of urbanization on breeding bird community structure in southwestern desert habitats. Condor 1989; 91: 416–428.
79. Ortega-Álvarez R, MacGregor-Fors I, Living in the big city: Effects of urban land-use on bird community structure, diversity, and composition. Landsc. Urban. Plan. 2009; 90: 189-195.
80. Korányi D, Gallé R, Donkó B, Chamberlain DE, Batáry P, Urbanization does not affect green space bird species richness in a mid-sized city. Urban Ecosyst. 2020; 1-12.

81. Park CR, Lee WS, Relationship between species composition and area in breeding birds of urban woods in Seoul, Korea. Landsc. Urban. Plan. 2000; 51: 29-36.
82. Murgui E, Valentin A, Relationships between the characteristics of the urban landscape and the introduced bird community in the city of Valencia (Spain). Ardeola. Int. J. Ornithol. 2003; 50: 201–214
83. Borcard D, Legendre P, Avois-Jacquet C, Tuomisto H, Dissecting the spatial structure of ecological data at multiple scales. Ecology 2004; 85: 1826-1832.
84. Chang LW, Zelený D, Li CF, Chiu ST, Hsieh CF, Better environmental data may reverse conclusions about niche-and dispersal-based processes in community assembly. Ecology 2013; 94: 2145-2151.
85. Johnson MD, Measuring habitat quality: a review. Condor 2007; 109: 489-504.

Supplementary Material:

**S1**. Correspondence analysis (a) to examine ecological space of bird species in Texas during the summer of 2013 and 2017, and correlation matrix (b) of significant PCAs and PCNMs.

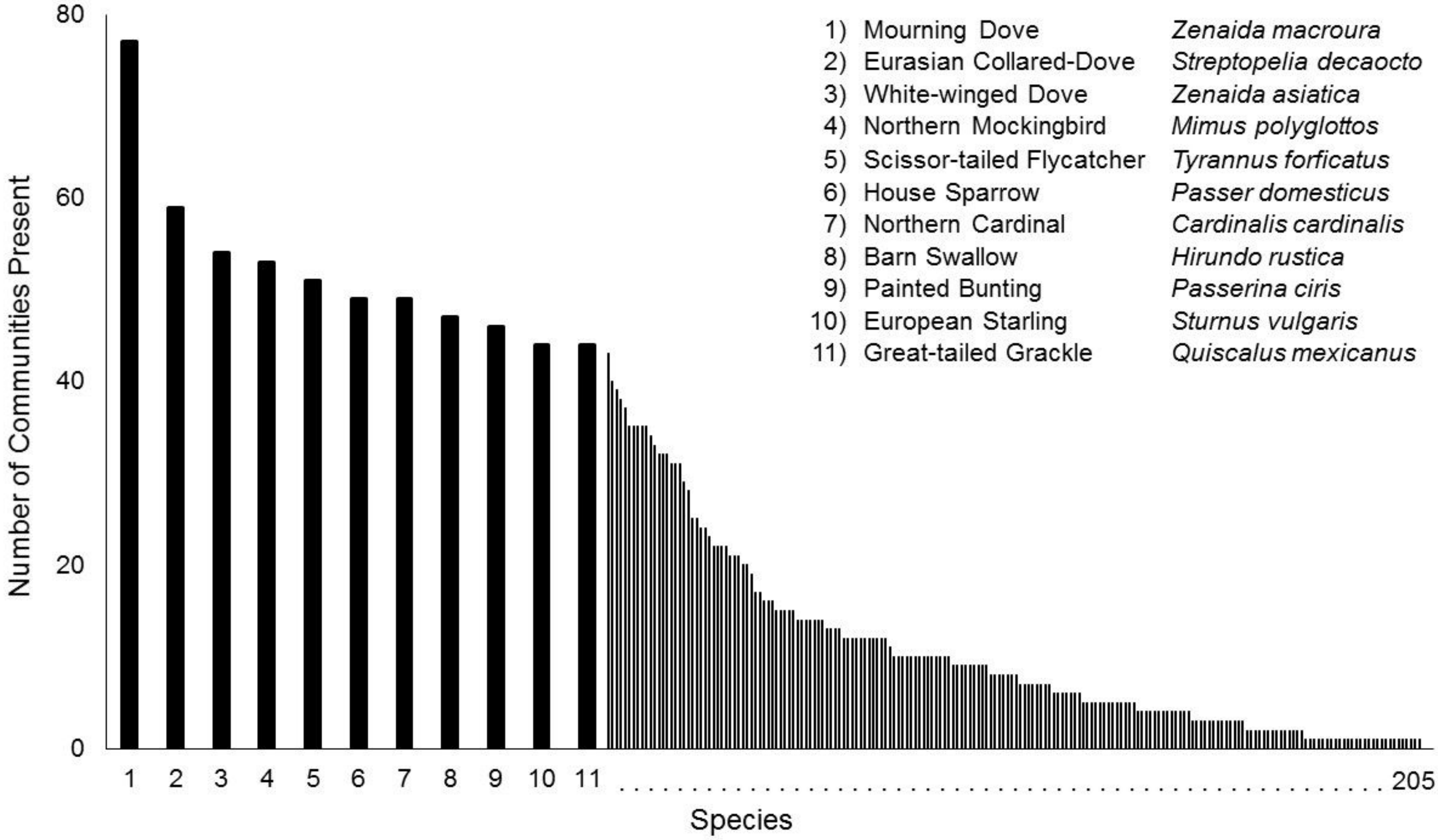

80
60
40
20
0
Number of Communities Present
1 2 3 4 5 6 7 8 9 10 11 ........ 205
Species
1) Mourning Dove *Zenaida macroura*
2) Eurasian Collared-Dove *Streptopelia decaocto*
3) White-winged Dove *Zenaida asiatica*
4) Northern Mockingbird *Mimus polyglottos*
5) Scissor-tailed Flycatcher *Tyrannus forficatus*
6) House Sparrow *Passer domesticus*
7) Northern Cardinal *Cardinalis cardinalis*
8) Barn Swallow *Hirundo rustica*
9) Painted Bunting *Passerina ciris*
10) European Starling *Sturnus vulgaris*
11) Great-tailed Grackle *Quiscalus mexicanus*

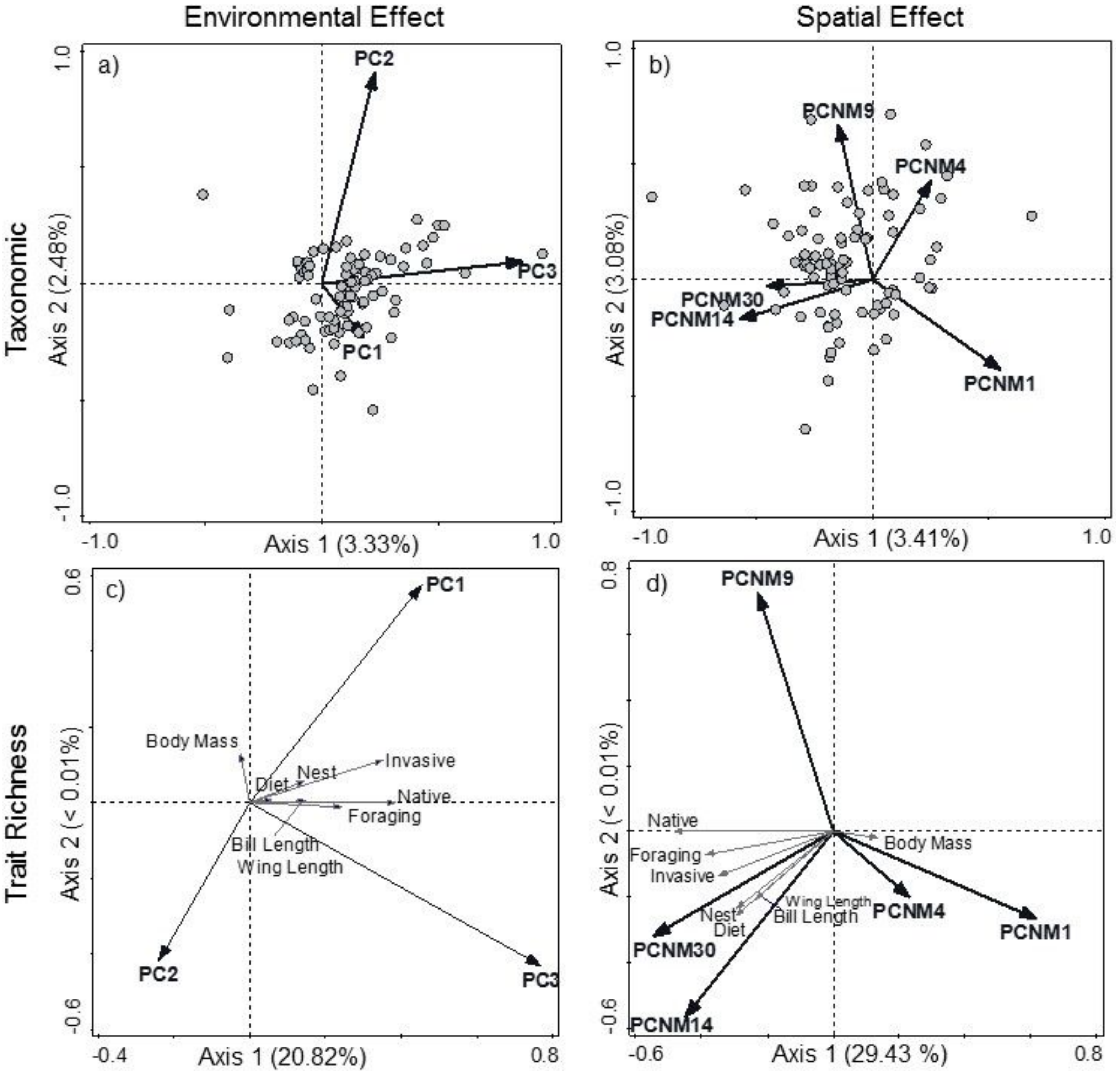

Environmental Effect
Spatial Effect
Taxonomic
Trait Richness
a)
PC2
PC3
PC1
Axis 2 (2.48%)
Axis 1 (3.33%)
1.0
-1.0
b)
PCNM9
PCNM4
PCNM30
PCNM14
PCNM1
Axis 2 (3.08%)
Axis 1 (3.41%)
c)
PC1
Body Mass
Invasive
Nest
Diet
Native
Foraging
Bill Length
Wing Length
PC2
PC3
Axis 2 (< 0.01%)
Axis 1 (20.82%)
0.6
-0.6
-0.4
0.8
d)
PCNM9
Native
Body Mass
Foraging
Invasive
Wing Length
Nest
Bill Length
Diet
PCNM4
PCNM1
PCNM30
PCNM14
Axis 2 (< 0.01%)
Axis 1 (29.43 %)

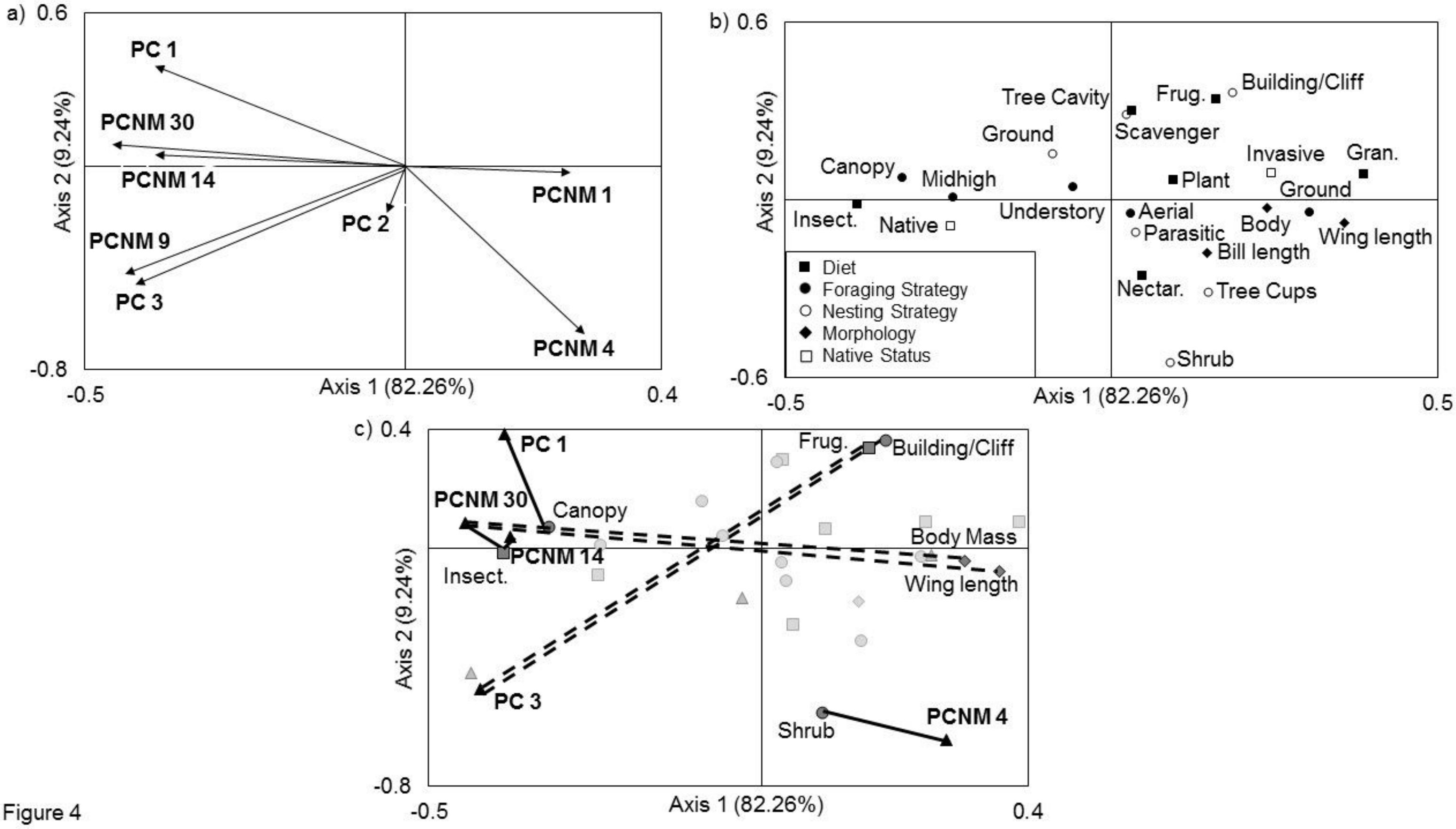
a)
0.6
-0.8
-0.5
0.4
Axis 1 (82.26%)
Axis 2 (9.24%)
PC 1
PCNM 30
PCNM 14
PCNM 9
PC 3
PC 2
PCNM 1
PCNM 4
b)
0.6
-0.6
-0.5
0.5
Axis 1 (82.26%)
Axis 2 (9.24%)
Tree Cavity
Frug.
Building/Cliff
Scavenger
Ground
Invasive
Gran.
Canopy
Midhigh
Plant
Ground
Understory
Insect.
Native
Aerial
Parasitic
Body
Wing length
Bill length
Nectar.
Tree Cups
Shrub
Diet
Foraging Strategy
Nesting Strategy
Morphology
Native Status
c)
0.4
-0.8
-0.5
0.4
Axis 1 (82.26%)
Axis 2 (9.24%)
PC 1
Frug.
Building/Cliff
PCNM 30
Canopy
Body Mass
PCNM 14
Insect.
Wing length
PC 3
PCNM 4
Shrub

Figure 4

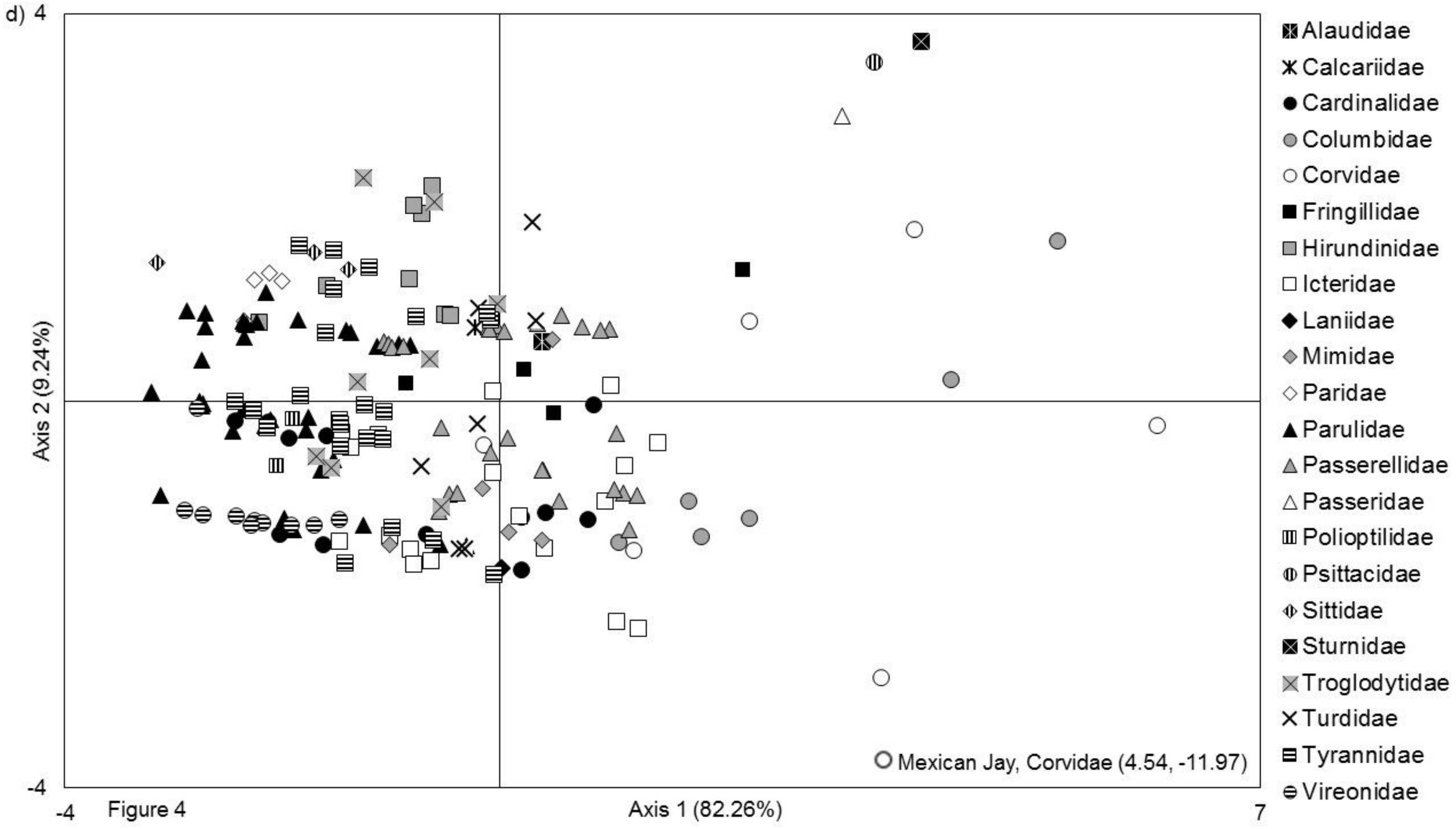
d)
4
-4
-4
7
Axis 2 (9.24%)
Axis 1 (82.26%)
Figure 4
Mexican Jay, Corvidae (4.54, -11.97)
Alaudidae
Calcariidae
Cardinalidae
Columbidae
Corvidae
Fringillidae
Hirundinidae
Icteridae
Laniidae
Mimidae
Paridae
Parulidae
Passerellidae
Passeridae
Polioptilidae
Psittacidae
Sittidae
Sturnidae
Troglodytidae
Turdidae
Tyrannidae
Vireonidae

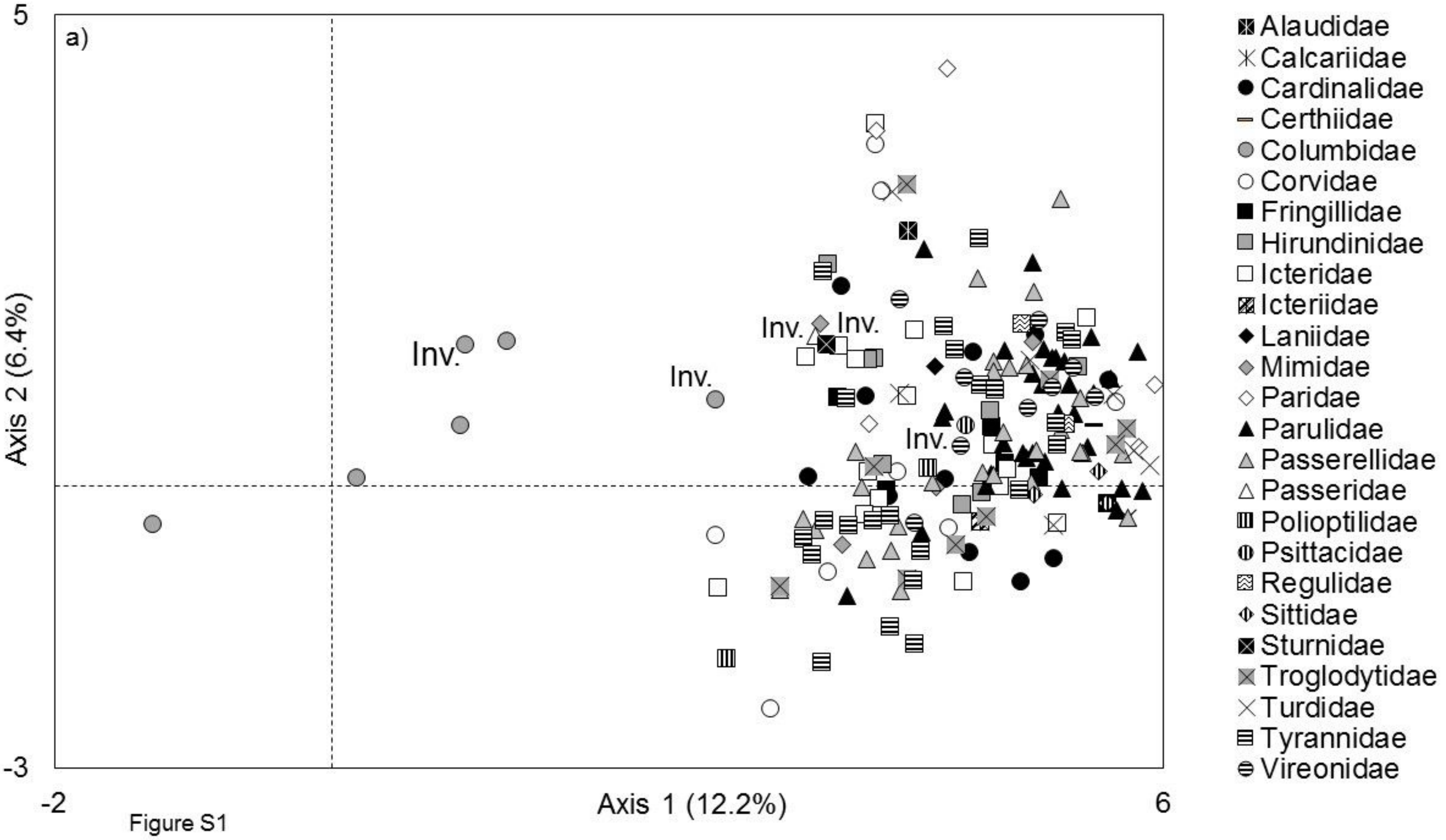
a)
5
-3
-2
6
Axis 1 (12.2%)
Axis 2 (6.4%)
Inv.
Inv.
Inv.
Inv.
Inv.
Alaudidae
Calcariidae
Cardinalidae
Certhiidae
Columbidae
Corvidae
Fringillidae
Hirundinidae
Icteridae
Icteriidae
Laniidae
Mimidae
Paridae
Parulidae
Passerellidae
Passeridae
Polioptilidae
Psittacidae
Regulidae
Sittidae
Sturnidae
Troglodytidae
Turdidae
Tyrannidae
Vireonidae

Figure S1

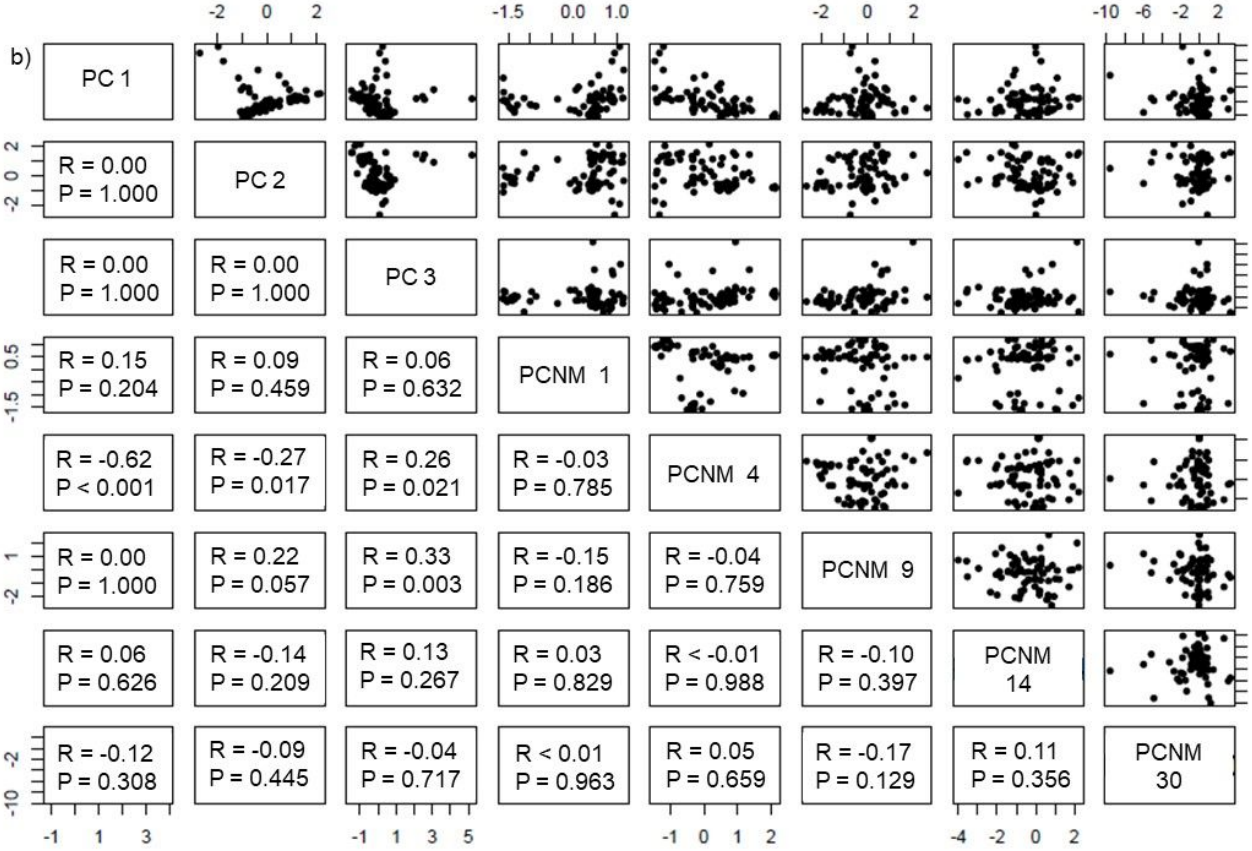

b)
PC 1
PC 2
PC 3
PCNM 1
PCNM 4
PCNM 9
PCNM 14
PCNM 30
R = 0.00 P = 1.000
R = 0.00 P = 1.000
R = 0.00 P = 1.000
R = 0.15 P = 0.204
R = 0.09 P = 0.459
R = 0.06 P = 0.632
R = -0.62 P < 0.001
R = -0.27 P = 0.017
R = 0.26 P = 0.021
R = -0.03 P = 0.785
R = 0.00 P = 1.000
R = 0.22 P = 0.057
R = 0.33 P = 0.003
R = -0.15 P = 0.186
R = -0.04 P = 0.759
R = 0.06 P = 0.626
R = -0.14 P = 0.209
R = 0.13 P = 0.267
R = 0.03 P = 0.829
R < -0.01 P = 0.988
R = -0.10 P = 0.397
R = -0.12 P = 0.308
R = -0.09 P = 0.445
R = -0.04 P = 0.717
R < 0.01 P = 0.963
R = 0.05 P = 0.659
R = -0.17 P = 0.129
R = 0.11 P = 0.356
